# Microbial Antagonism Toward *Botrytis* Bunch Rot of Grapes in Multiple Field Tests Using One *Bacillus ginsengihumi* Strain and Formulated Biological Control Products

**DOI:** 10.3389/fpls.2019.00105

**Published:** 2019-02-11

**Authors:** Carlos Calvo-Garrido, Jean Roudet, Nicolas Aveline, Ludivine Davidou, Sévérine Dupin, Marc Fermaud

**Affiliations:** ^1^SAVE, INRA, Institut National de la Recherche Agronomique, Bordeaux Science Agro, ISVV 33882, Villenave d'Ornon, France; ^2^Institut Français de la Vigne et du Vin–Vinopôle Bordeaux-Aquitaine, Blanquefort, France; ^3^Chambre d'Agriculture de la Gironde (CA33)–Service Vigne et Vin. CS 20115, Blanquefort, France

**Keywords:** gray mold, biocontrol, *Vitis vinifera*, disease support system, biological control agents, wine grapes

## Abstract

*Botrytis* bunch rot (BBR), caused by the necrotrophic fungus *Botrytis cinerea*, is a major disease of wine and table grapes worldwide. Due to negative effects of pesticides on the environment and human health, alternative control strategies against BBR, such as biological control agents (BCAs), are required to produce high-quality grapes and wines with high standards of food safety. However, few biological control products against BBR are available, and their efficacy is sometimes variable. This study aimed to evaluate and compare (1) the efficacy of new bacterial BCA strains developed at INRA Bordeaux and (2) the BBR reductions achieved by commercial biocontrol products that are already registered or close to being registered. During three consecutive seasons, 10 field experiments were established in six different experimental vineyards in southwestern France. Spray applications were performed at key phenological stages (five or six during the season), or at high BBR-risk periods late in the season according to a Disease Risk Index model. At harvest, BBR incidence and severity (% of symptomatic berries per bunch) were visually determined. The experiments included four bacterial strains at an early experimental stage, particularly *Bacillus ginsengihumi* (S38). Nine commercial BCA products were also tested, including *Bacillus subtilis, Bacillus amyloliquefaciens, Aureobasidium pullulans, Ulocladium oudemansii*, and *Candida sake*. Among the four experimental bacterial strains, only *B. ginsengihumi* S38 significantly controlled the BBR, presenting reductions in the average severity ranging from 35 to 60%, compared to untreated control, throughout the three seasons. Several commercial BCAs achieved significant reductions in BBR severity ranging from 21 to 58%, although not in every trial. The treatments that achieved higher reductions in severity rates were based on *C. sake* (45%), *B. subtilis* (54%), and *B. amyloliquefaciens* (58%). The efficacy of those BCAs was consistent throughout the studied seasons. The results confirmed the suitability of several biological control products under the conditions in vineyards in southwestern France, while also highlighting the good performance of the novel experimental BCA *B. ginsengihumi* S38 strain, which achieved similar control rates to the products registered for commercial use. The major factors involved in the variability in the results are also discussed.

## Introduction

In many economically important agricultural and horticultural crops worldwide, *Botrytis cinerea* Pers.:Fr. is a major necrotrophic fungal pathogen causing gray mold (Elad et al., 2016). This disease is serious and leads to considerable yield and quality losses in field production and storage (Sharma et al., 2009), especially in wine grape production (Ky et al., 2012; Steel et al., 2013). Although various anti-*Botrytis* synthetic fungicides are available, their use in pre- or post-harvest conditions is not considered sustainable because of (i) the frequent appearance of resistant strains (Leroux, 2004; Walker et al., 2013; Hahn, 2014) and (ii) the adverse effects of the fungicides on the environment and human health (Komárek et al., 2010). This last issue is being investigated more thoroughly since some major anti-Botrytis synthetic active ingredients are often considered to present risks to human health and are frequently detected in different food products, including wine.

The residues of synthetic anti-Botrytis fungicides drastically affect food quality. Three of these fungicides, commonly used in vineyards (active ingredients: pyrimethanil, cyprodinil, and fludioxonil), have shown marked toxic effects on human glial, androgenic, or neuronal cells (Orton et al., 2011; Coleman et al., 2012). Health risk due to these anti-Botrytis fungicides emerge from frequent human and environmental exposure to these chemicals, particularly because of the presence of residues in wines worldwide. Most of the specific anti-*Botrytis* synthetic fungicides are responsible for the possible contamination of commercial wines. Esteve-Turrillas et al. (2016) reported that 44% of the international wines sampled contained at least one of the five anti-*Botrytis* active ingredients they targeted. The same fungicide residues also have been reported as often present in other international wines (Economou et al., 2009; Cuš et al., 2013; Pelajić et al., 2016). Therefore, the use of anti-*Botrytis* pesticides represents an issue of worldwide relevance, with potential implications for consumer health and international trade. High presence of anti-Botrytis fungicides in wines may be due to its regular use close to harvest, showing a considerable transfer rate from grapes to wine, because of the physico-chemical characteristics of the fungicides. A study conducted on French wines showed that 30% of the overall analyzed pesticides in grapes were transferred to wines, and among the residues found in European conventional wines, four compounds were specific anti-*Botrytis* fungicides, i.e., boscalid, pyrimethanil, fludioxonil, and iprodione (PAN Europe, 2008).

*Botrytis cinerea* is a high-risk plant pathogen due to its high genetic variability, short life cycle, and prolific reproduction (De Miccolis Angelini et al., 2016; Elad et al., 2016). These characteristics also favor the development of fungicide resistance, which reduces the efficacy of specific synthetic fungicides. Resistance phenomena by *B. cinerea* have been analyzed in numerous studies on different crops (Zhao et al., 2010; Walker et al., 2013; Hahn, 2014; Fillinger and Walker, 2016). Moreover, chemical control in agriculture *sensu lato* is also under drastic regulatory constraints in some countries. For example, European Union (EU) policy is moving toward a marked decrease in pesticide use and the development of sustainable agriculture based on the Integrated Pest Management (IPM) principles mandated by the Directive 128/2009/EC (Lamichhane et al., 2015; Lamichhane, 2017).

In the context of multiple constraints on synthetic pesticide use, the development of methods complementary to chemical control, such as the use of non-pathogenic microorganisms as biological control agents (BCAs), is increasingly considered as promising alternatives. However, only a few commercial products, based on fungal or bacterial genera are available in Europe for the biological control of BBR (Nicot et al., 2016). Currently in France (Index-Acta-phytosanitaire, 2017), six biocontrol products are registered for viticulture: three products based on the microbial antagonists *Bacillus subtilis, Aureobasidium pullulans*, and *Bacillus amyloliquefaciens* (Serenade Max®, Botector®, and Amylo-X®, respectively) and three based on Potassium Bicarbonate, essential oils (eugenol, thymol, and geraniol) and gibberellic acid (Armicarb®, Mevalone®, and Berelex 40 SG®). However, the field efficacy against BBR of these different products, as well as the efficacy consistency between seasons, different climatic seasonal patterns, and/or local agronomic conditions is still questioned, especially by grapevine growers and technicians. Similarly, some published scientific articles have also noted the lack of efficacy in viticulture of some of these products, even when numerous treatments were applied within a season (Rotolo et al., 2018). This variability justifies the need for reliable vineyard studies to investigate these points and to include recently registered biocontrol products in these studies.

Biological control of *B. cinerea* is thoroughly investigated in economically important crops such as tomatoes, strawberries, and grapes since more than 30 years (Sylla et al., 2015; Haidar et al., 2016b; Marín et al., 2016; Nicot et al., 2016; Passera et al., 2017). In INRA Nouvelle-Aquitaine-Bordeaux (UMR SAVE), recent research has evaluated the efficacy of up to 46 bacterial strains against *B. cinerea* infection in grapevines, using both laboratory biotests and vineyard experiments; (Haidar et al., 2016a; Calvo-Garrido et al., 2018). Among these strains, the *Bacillus ginsengihumi* S38 strain, originally isolated from grapevine wood (Bruez et al., 2015), was shown to be the most effective with a high potential for BBR biocontrol and future development in vineyards. First, the results for the S38 grapevine origin and field population dynamics (Calvo-Garrido et al., 2018) showed the ability of this strain to survive in the vineyard ecosystem. Because of its ability for field survival, this S38 strain was confirmed as being potentially properly adapted for biological control in vineyards, according to other studies (Demoz and Korsten, 2006; Gotor-Vila et al., 2017; Mutlu et al., 2018). Second, the laboratory selection process was based on three key biocontrol features: (1) high overall strain efficacy from *in vivo* grapevine biotests (Haidar et al., 2016a), (2) potentially highly efficient mode(s) of action (MoA), and (3) high or intermediate survival ability under two contrasting simulated climatic conditions (Calvo-Garrido et al., 2018). As widely described for other *Bacillus* species (Ongena and Jacques, 2008; Baruzzi et al., 2011; Ambrico and Mario, 2017), the S38 strain MoA arose more from metabolite production than from nutrient competition (Calvo-Garrido et al., 2018). This finding is also consistent with the literature because *Bacillus* species, notably the *B. subtilis* strains, are considered of prime importance in the biological control of grapevine diseases (Ongena and Jacques, 2008; Di Francesco et al., 2016; Haidar et al., 2016b; Nicot et al., 2016; Sawant et al., 2016; Pertot et al., 2017b). The selection process of candidate strains was validated by a one-season vineyard trial showing that four or five S38 applications during the season significantly reduced the incidence of BBR 72 to 75% compared to the control. Moreover, the use of a Disease Risk Index (DRI) model for positioning the late-season BCA strain applications also led to a significant reduction in BBR at harvest (Calvo-Garrido et al., 2018). However, field efficacy of the S38 strain must be confirmed in repeated multi-year trials, whereas other bacterial strains, pre-selected in the cited study, may be introduced in field experiments for evaluation of their potential activity against BBR.

Biocontrol strategies are generally known as highly variable in their field efficacies (Nicot et al., 2011; Pertot et al., 2017a). Some major variation factors may affect the field efficacy of the *B. ginsengihumi* S38 strain: (i) the season effect, due to annual climatic conditions; (ii) adapted timing and frequency of the BCA applications; (iii) BCA formulation, including or not BCA culture supernatant; and (iv) soil conditions and cultural practices.

The objectives of the study were, first, to thoroughly evaluate the efficacy of *B. ginsengihumi* S38 and other seven bacterial antagonists at a developmental stage, during three growing seasons in the Bordeaux area. Second, to test the efficacy of novel BCA application strategies based on a BBR epidemiological risk model and on BCA combinations. Third, during the same 3-year period, we aimed to test the efficacy of commercial BCA products, to generate practical information for growers on the performance and efficacy variability of those antagonists compared to the experimental *B. ginsengihumi* S38 strain.

## Materials and Methods

### Experimental Field Sites

All field experiments were conducted in two different experimental platforms, each consisting of several field sites. The first platform, consisting of two INRA-owned vineyards, was used for testing control strategies that included the experimental bacterial strains recently developed by INRA (UMR SAVE, Bordeaux). The first INRA site, “GF” (cv. Merlot noir), is in Villenave d'Ornon, near Bordeaux. The vine stocks were planted in 1991 on a typical gravel soil and were grafted on “101-14” rootstock. The planting density was ~5,350 vines ha^−1^. This plot was used in the 2016 and 2017 growing seasons. The second INRA site, “CHS” (cv. Semillon blanc), is in Cadaujac, near Bordeaux. The vine stocks were planted in 2004, grafted on Fercal rootstock, with a density of 4,348 vines ha^−1^. This plot was used in the 2015 and 2016 growing seasons.

The second major experimental platform was managed by the Bordeaux viticulture service of the Agriculture Chamber of Gironde (CA33) and the French Institute of Grapevine and Wine (IFV). On this platform, we mostly tested products registered for commercial use and/or close to being registered. Four vineyard sites were used. The first site, “Avensan” (cv. Merlot noir), is near Margaux (Margaux Appellation of Origin), ~20 km north of Bordeaux. It was planted in 2009, at a density of 5,500 vines ha^−1^ on clayey-sand soil and 101-14 rootstock. This site was only used during the 2015 season. The second site, “StYzan” (cv. Merlot noir), is in the Medoc grapevine growing area, ~60 km north of Bordeaux (Medoc Appellation of Origin). The planting density was 4,500 vines ha^−1^ on clayey-silt soil. It was planted in 2000 on Fercal rootstock. This vineyard was used in the 2016 and 2017 seasons. The third field site, “Montagne” (cv. Merlot noir), is at the Viticulture School of Libourne-Montagne (Montagne-Saint-Emilion Appellation of Origin), ~50 km east of Bordeaux. The site was planted on a silty sand soil in 1992 with 3309C rootstock at a planting density of 6,060 vines ha^−1^. This vineyard was used in the 2015 and 2016 seasons. The fourth plot, “Langoiran” (cv. Cabernet Sauvignon), was used in 2017 only. This plot is located ~25 km south-east of Bordeaux, in the Entre-deux-mers winegrowing region, and it was planted in 2003 with 101-14 rootstock in a clayey gravel soil at 5,000 vines ha^−1^.

All vineyards were trained in a double Guyot system, typical of the Bordeaux region. In the IFV-CA33 vineyards, in 2016 and 2017, the grapevines were manually or mechanically leaf-plucked along one side of the row at the pea-size stage. Otherwise, no leaf removal was carried out. If a plot was mechanically leaf-plucked, an evaluation was conducted to ensure that bunches were not damaged by the engine.

The experimental vineyards were not treated with any specific anti-*Botrytis* fungicide, with the exception of “StYzan” in 2015, in which a fungicide application (37.5% cyprodinil + 25% fludioxonil, at 1 kg ha^−1^) was carried out in the stage between pea-size and bunch closure (24/06/15), due to a technical error in vineyard management. Phytosanitary protection against powdery mildew, downy mildew and grape berry moth was conducted following the criteria of each vineyard manager.

### Biologically Based Treatments

#### Experimental Antagonistic Bacterial Strains

The experimental bacterial isolates used were originally isolated from grapevine tissues (Martins et al., 2013; Bruez et al., 2015) and were maintained in the collections of either INRA Bordeaux-Aquitaine (UMR SAVE) or “Biological Resources Center for Enology” (University of Bordeaux and Bordeaux Polytechnic Institute). These BCA strains were characterized in previous studies (Haidar et al., 2016a,c). Accordingly, out of 46 candidate strains, a short list of 10 BCA strains with high biological control potential was developed, and it included *Enterobacter cowanii* S22, *Bacillus ginsengihumi* S38, *Paenibacillus sp*. S18, *Enterobacter sp*. S23, and *Pantoea agglomerans* S6. The materials and methods used for the storage, culture in liquid media, centrifugation and adjustment of concentrated suspensions were previously described by Calvo-Garrido et al. (2018). The detailed list of strains used is summarized in Table 1.

**Table 1:** Biological control agents, adjuvants and natural products applied in the field studies.

**Active ingredient**	**Commercial product name**	**Applied dose**	**Manufacturer**	**Registration status**
***MICROORGANISMS*** **(EXPERIMENTAL)**				
*Bacillus ginsengihumi* S38	Developmental stage	5 x 10^7^ CFU ml^−1^	INRA-Bordeaux, France	–
*Enterobacter cowanii* S22	Developmental stage	5 x 10^7^ CFU ml^−1^	INRA-Bordeaux, France	–
*Paenibacillus* sp. S18	Developmental stage	5 x 10^7^ CFU ml^−1^	INRA-Bordeaux, France	–
*Enterobacter* sp. S23	Developmental stage	5 x 10^7^ CFU ml^−1^	INRA-Bordeaux, France	–
*Pantoea agglomerans* S6	Developmental stage	5 x 10^7^ CFU ml^−1^	INRA-Bordeaux, France	–
*Bacillus* sp. IP	Developmental stage	4 kg ha^−1^	Ital Pollina, Italy	–
*Candida sake* CPA-1	Developmental stage	3-4 x 10^7^ CFU ml^−1^	IRTA-Lleida, Spain	Registered in Spain as plant health enhancer
*Trichoderma* sp. IP	Developmental stage	2.5 kg ha^−1^	Ital Pollina, Italy	Registration in process (France)
***MICROORGANISMS*** **(COMMERCIAL)**				
*Bacillus subtilis* QST713	Serenade Max®	2 kg ha^−1^	Bayer SAS Cropsicence, France	Registered in France, Biocontrôle list
*Bacillus amyloliquefaciens* subsp. *plantarum* strain D747	Amylo-X®	2,5 kg ha^−1^	Certis Europe, Nederlands	Registered in France, Biocontrôle list
*Aureobasidium pullullans* strains DSM14940 and DSM 14941	Botector®	0.4 kg ha^−1^	Bio-Ferm, Austria	Registered in France, Biocontrôle list
*Ulocladium oudemansii* HRU3	Botryzen®	4 kg ha^−1^	Botryzen Ltd, New Zealand	Registered in New Zealand
*Bacillus subtilis* IAB/BS03	Fungisei®	3 l ha^−1^	Seipasa, Spain	Registered in USA Registration in process (Spain)
**NATURAL PRODUCTS**				
Fatty acids	MidiZen®	3% (v/v)	Botryzen Ltd, New Zealand	Registered in New Zealand
Chitosan	ArmourZen®	1% (v/v)	Botryzen Ltd, New Zealand	Registered in New Zealand
Fatty acid emulsion	Fungicover®	1% (v/v)	BioDurcal, Spain	Registered in Spain as plant health enhancer
**ADJUVANT**				
Synthetic latex	Sticman®	0.14% (v/v)	Agridyne, De Sangosse, France	Registered in France

Concentrated bacterial suspensions used for field experiments contained the culture supernatant as liquid matrix. However, in 2016 and 2017 seasons, we also included non-supernatant treatments (“NS”) in which the bacteria cells were resuspended only in phosphate buffer (Tables 2, 3; pH 6.5; KH2PO4 0.2 mol l^−1^, 70 ml; K2HPO4 0.2 mol l^−1^, 30 ml and deionized water, 300 ml).

**Table 2:** *Botrytis* bunch rot control strategies evaluated at “CHS” experimental field site including phenological stages, application dates and efficacy results.

**Strategy**	**Treatment^a^**	**A 10% flowering**	**A+ 100% flowering**	**B Pre bunch closure**	**C 10% veraison**	**D 21 days before harvest**		**% Incidence BBR^c^**	**% Severity BBR**	**% Severity reduction compared to control**
2015		10/06^b^	18/06	10/07	07/08	03/09		15/09		
							*p*-value^d^	0.106	0.300	
Untreated	Control	—	—	—	—	—		51.9	13.4	
Full season strategy	S38	+	+	+	+	+		60.2	10.7	20
	S22	+	+	+	+	+		44.2	6.9	48
	ADJ	+	+	+	+	+		45.5	6.15	54
Single application– C stage	Single C-S38	—	—	—	+	—		67.2	10.9	19
	Single C-S22	—	—	—	+	—		73.1	12.8	4
Single application– D stage	Single D-S38	—	—	—	—	+		40.8	5.4	59
	Single D-S22	—	—	—	—	+		61.2	13.0	3
2016		13/06	22/06	12/07	17/08	31/08		19/09		
							*p*-value	0.315	0.278	
Untreated	Control	—	—	—	—	—		75.3	20.3	
Full season	ADJ	+	+	+	+	+		84.9	21.7	−7
	S38 (NS)	+	+	+	+	+		79.9	18.5	9
	S38	+	+	+	+	+		78.2	13.9	32
	S38 (NS-NoADJ)	+	+	+	+	+		67.0	10.4	49
	S38 (NoADJ)	+	+	+	+	+		73.8	12.2	40

a *Treatments consisted of spray applications (+) of biological control agents at five key stages of grapevine phenology (“Full Season” strategy), unless other strategy is defined for a particular treatment. Control, Untreated; Single, One single application at C or D phenological stages; S38, Bacillus ginsengihumi S38 strain at 5 x 10^7^ CFU ml^−1^ + ADJ; S22, Enterobacter cowanii S22 strain at 5 x 10^7^ CFU ml^−1^ + ADJ; ADJ, Sticman® adjuvant at 0.14% (v/v); (NS), Non Supernatant in the bacterial formulation; NoADJ, No adjuvant (ADJ) in the treatment mixture*.

b *Spray application dates. Applications were carried out with a motorized back sprayer until runoff*.

c *Incidence and severity of Botrytis bunch rot (BBR) were visually assessed at commercial harvest dates. Values are means of four replicate plots*.

d *p-values correspond to the “Treatment” effect in multifactorial ANOVA*.

**Table 3:** *Botrytis* bunch rot control strategies evaluated at “GF” experimental field site including phenological stages, application dates and efficacy results.

**Strategies**	**Treatment^a^**	**A 10% flowering**	**A+ 100% flowering**	**B Pre bunch closure**	**C 10% veraison**	**D 21 days before harvest**		**% Incidence BBR^c^**	**% Severity BBR**	**% Severity reduction compared to control**
2016		01/06 ^b^	08/06	13/07	09/08	07/09		06/10		
							*p*-value^d^	0.184	0.313	
Untreated	Control	—	—	—	—	—		53.4	4.3	
Full season	S38 (NS)	+	+	+	+	+		53.0	4.4	−2
	S38	+	+	+	+	+		42.8	3.0	30
	S18	+	+	+	+	+		70.5	5.7	−31
	S23	+	+	+	+	+		48.5	2.7	37
	Cocktail	+	+	+	+	+		60.5	4.3	0.2
	ADJ	+	+	+	+	+		65.9	5.3	−22
Early season	ES – S38	+	+	+	—	—		45.0	2.5	42
Late season DRI based	DRI – S38	—	—	—	S38 - DRI output 2 applications			62.5	5.6	−28
	DRI – Cocktail	—	—	—	S38+S18+S23 2 applications			44.9	3.0	32
2017		01/06	10/06	04/07	26/07	23/08		15/09		
							*p*-value	0.102	0.042^*^	
Untreated	Control	—	—	—	—	—		56.7	9.9a	
Full season	S38	+	+	+	+	+		42.0	4.9c	51
	S38 (NS)	+	+	+	+	+		34.7	4.0bc	60
	S6	+	+	+	+	+		49.0	8.8ab	11
	ADJ	+	+	+	+	+		44.4	6.3abc	36
Early Season	ES - S38	+	+	+	—	—		40.9	7.9ab	20
Late season DRI based strategy	DRI - S38	—	—	—	S38 - DRI output 3 applications			52.4	7.98ab	20

a *Treatments consisted of spray applications (+) of biological control agents at five key stages of grapevine phenology (“Full Season” strategy), unless other strategy is defined for a particular treatment. Control, Untreated; ES, “Early Season” strategy, three applications from flowering to pre-bunch closure; DRI, Applications following a modeled Disease Risk Index used to trigger sprays after veraison; S38, Bacillus ginsengihumi S38 strain at 5 x 10^7^ CFU ml^−1^ + ADJ; S18, Paenibacillus sp. S18 strain at 5 x 10^7^ CFU ml^−1^ + ADJ; S23, Enterobacter sp. S23 strain at 5 x 10^7^ CFU ml^−1^ + ADJ; S6, Pantoea agglomerans S6 strain at 5 x 10^7^ CFU ml^−1^ + ADJ; Cocktail, S38+S18+S23+ADJ; ADJ, Fungicover® natural product at 1 % (v/v); (NS), Non Supernatant in the bacterial formulation*.

b *Spray application dates. Applications were carried out with a motorized back sprayer until runoff*.

c *Incidence and severity of Botrytis bunch rot (BBR) were visually assessed at commercial harvest dates. Values are means of five replicate plots*.

d *p-values correspond to the “Treatment” effect in multifactorial ANOVA*.

* *Significant differences detected; Mean values connected by same letters are not significantly different (p < 0.05)*.

Concentrated suspensions were stored at 15 °C in the dark until field applications. Just before application, concentrated suspensions were diluted 1:14 ± 2 in tap water. An additive was added into the mixture to favor the persistence of the bacterial cells on the grapevine bunches. The commercial adjuvant Sticman® was used in both “CHS” trials in 2015 and 2016 because it had shown significantly improved BCA cell adherence in an earlier laboratory experiment (Calvo-Garrido et al., 2018). The natural product Fungicover® was used at a low dose (1% v/v) compared to the full dose (3–5% v/v) recommended for anti-Botrytis protection (BioDúrcal, 2008, 2010). This product was also demonstrated to favor cell adherence of several INRA bacterial candidates in preliminary studies (data not shown) and to improve the survival of other BCAs, such as *C. sake* (Calvo-Garrido et al., 2014c).

#### Commercial Biological Control Agents (BCA) and Natural Products

The field trials conducted in the IFV-CA33 experimental platform aimed to compare the efficacy of commercial BCAs in the typical conditions of oceanic climate and grapevine cultivars in vineyards in southwestern France. Biological control products were selected and included (i) products already registered and commercialized in France; (ii) products registered in other countries (New Zealand, Spain, and the United States); (iii) products close to registration in France, thanks to the contribution of phytosanitary firms developing BCA products; and iv) other BCAs still in a developmental stage by private manufacturers or research laboratories. The details concerning the product origin, species of the BCA microorganism included, legal status, and applied dose are summarized in Table 1. In addition to including these microbial products, Table 1 also includes a list of non-microbial products that are used as adjuvants for BCA applications (as described above) or are used as anti-*Botrytis* natural products in a combinational strategy. In the “BZ strategy” treatment, the BCA *U. oudemansii* fungus was combined with applications of natural products in the middle and late growing season (Table 3). The doses applied for either the natural products or the microbial products tested were in accordance with manufacturer recommendations (Table 1).

#### Experimental Design and Control Strategies Tested

The experimental design in every field plot was in randomized blocks, where each replicate unit consisted of seven to ten adjacent vines with the first and last vines used as buffer vines. Each treatment included four replicate plots in “CHS” (2015 and 2016), “Avensan” (2015), and “Montagne” (2015) and five replicates in “GF” (2016 and 2017), “Montagne” (2016), “StYzan” (2016 and 2017), and “Langoiran” (2017).

The field treatments consisted of spray programs with applications at key phenological stages, as presented in Tables 2–5 for the experiments conducted in the “CHS,” “GF” and IFV-CA33 platform sites, respectively. In every experimental site, the biocontrol treatments were always compared to untreated control plots. Details of concentrations, active ingredients applied, and the timing of spray applications are summarized in Tables 1–5. The growing season was divided into six key phenological stages, classically used in *B. cinerea* control strategies in vineyards worldwide, from flowering to harvest: A = Flowering (10% cap fall; BBCH = 61), A+ = Flowering (100% cap fall; BBCH = 69), B = Pre-bunch closure (BBCH = 77), C = 10% Veraison (BBCH = 81), C+ = Veraison + 21 days (BBCH = 85), and D = 21 days before harvest (BBCH = 85).

The first strategy, “Full season,” consisted of five or six applications, depending on the experiment. The second strategy, “Early season” (“ES”), consisted of three applications before the veraison stage. Finally, the third strategy consisted of applications late in the season, after the veraison stage and following determination of a Disease Risk Index (DRI), previously developed at INRA Bordeaux-Aquitaine (UMR SAVE). The DRI index gives a numeric value for the daily BBR risk in a specific location, based on mathematic modeling of *B. cinerea* infection of mature grape berries (Ciliberti et al., 2015). The calculation of daily values of BBR risk based on Temperature and Relative Humidity hourly data, as well as the decision rules used to trigger spray applications, were as described by Calvo-Garrido et al. (2018). Furthermore, specifically and only at the “GF” INRA plot in 2016 and 2017, a more advanced decision rule was tested. The same equation of the DRI daily index was used, but the DRI was multiplied by a weighted index “WI,” varying from 0 to 1, to account for the ontogenic resistance dynamics during berry maturation. The weighted index was calculated on the basis of the study of (Deytieux-Belleau et al. (2009) using the following equation (1):

(1) $$\text{WI}=4^{*}10^{-7}{\text{X}}^{2}+0.0003\text{X}+0.0988$$

Where X (in °C ^*^ days) is the summation of daily mean temperatures issued from a standard meteorological automatic station situated close to the INRA plot. The X thermal summation was initiated at mid-veraison following a precise visual assessment in the experimental field (08/08/2016 and 25/07/2017). The DRI threshold for initiating the first post-veraison application amounted to 7%, and this first application was also conditioned by field detection of first BBR symptoms in bunches (> 1% in incidence). Considering the following treatments, the DRI threshold was subsequently established at 12%, regardless of the development of BBR symptoms. For all the DRI applications in 2015, 2016, and 2017, the application dates allowed a minimal gap period of 10 days between two treatments.

In this study, the decision rules triggered the following: (i) three sprays in 2015 (“Montagne”: 18/08/15, 03/09/15 and 14/09/15; “StYzan”: 17/08/15, 26/08/15 and 11/09/15), (ii) one or three sprays in 2016 (“GF”: 13/09/16 and 30/09/2016; “Montagne”: 18/08/16, 09/09/16 and 13/09/16; “StYzan”: 11/08/16, 05/09/16 and 13/09/16), (iii) three or four sprays in 2017 (“GF”: 17/08/17, 31/08/17 and 11/09/17; “Langoiran”: 21/08/17, 01/09/17, 14/09/17; “StYzan”: 26/07/17, 17/08/17, 31/08/17 and 14/09/17).

#### Field Applications and BBR Assessment

Treatment mixtures were prepared just before application by blending products into the corresponding volume of tap water to be applied on the replicate plots at the required dose. Spray applications were always focused in the bunch zone of the vines and carried out with two different back sprayers, depending on the experimental platform. In the INRA platform, we used an electric backpack jet-sprayer (416 Li model, SOLO GmbH, Sindelfingen-Maichingen, Germany) to spray grape bunches until achieving runoff. For the IFV-CA33 platform, a pneumatic atomizer was used at a rate of 400 l ha^−1^ (SR 450 model; Stihl France, Torcy, France), with the following exceptions: first, the “BZ strategy” treatment was applied in 2016, in which the *U. oudemansii* applications were performed with an increased water rate of 500 l ha^−1^, and then, the “S38–600 jet” and “S38–600 jet DRI” treatments were applied in 2017, where an electric jet-sprayer (416 SOLO GmbH, Sindelfingen-Maichingen, Germany) was also used at 600 l ha^−1^ for efficacy comparable to that of pneumatic applications and the results from the other platform.

At the end of each growing season, according to the commercial harvest dates and depending on the grape cultivar, BBR incidence, and severity were assessed by visually rating 50 grape bunches per replicate plot. The incidence of BBR corresponded to the percentage of bunches with typical *B. cinerea* rot symptoms, and BBR severity was measured visually as the percentage of *B. cinerea* rotten berries per bunch. Average percentage of disease incidence and severity per replicate plot were then calculated for subsequent analysis. In addition, if detected, the percentages for incidence and severity of Downy mildew were also visually assessed at harvest in the same bunches, as described for BBR. Meteorological data were collected from automated weather stations placed near the experimental vineyards. Hourly measurements of temperature and relative humidity were recorded throughout the three seasons.

### Population Dynamics of Experimental Bacterial Strains

Populations of the experimental INRA bacterial strains were monitored in INRA experimental platform trials. The population monitoring focused on the promising strain *B. ginsengihumi* S38, although the *E. cowanii* S22 strain was also monitored in 2015 at the “CHS” site. During the 2015 and 2016 seasons, tissue samples (flowers or berries) were collected just before a spray treatment, two hours after each application and at harvest. In the 2017 experiment at the “GF” location, populations of bacterial isolates were only sampled three times: after the 100% flowering and the veraison applications, and at harvest.

At flowering sampling times (10% flowering and 100% flowering, BBCH stages 61 and 69, respectively), BCA populations were recovered from 2 g of floral organs collected randomly from eight inflorescences per replicate unit. Samples were then immersed in 20 ml of phosphate buffer. At pre-bunch closure (BBCH stage 75), 40 pea-sized berries, sampled randomly from 20 bunches per unit plot, were weighed and then immersed in 50 ml of phosphate buffer. After veraison (BBCH stage 83), 20 berries were sampled randomly from 10 bunches, weighed and then immersed in 50 ml of phosphate buffer. Following serial dilutions of the washing solution, aliquots of each replicate were plated in duplicate. Then, after 24 to 48 h of incubation at 27°C, colony counts were carried out, based on morphological recognition of the bacterial strains. Data of CFU ml^−1^ were finally expressed as CFU g^−1^ of sample.

### Statistical Analyses

Multifactorial ANOVA was used for comparison of the BBR incidence and BBR severity data from the different locations and treatments. Mean values of BBR severity from the “GF” field site in 2017 were square root transformed prior to analysis. The percentage of downy mildew incidence was used as a significant covariable for the ANCOVA analysis in 2016 at the “GF” vineyard. Comparisons of treatment means with associated untreated control data were performed using the Least Significant Difference test. CFU data for the bacterial populations were log transformed before ANOVA, and the Tukey honestly significant difference (HDS) *post hoc* test was used for population values at a specific sample time. All tests were performed using the software JMP® Pro 12.0.1 (SAS Institute). Significant differences were analyzed at *p* < 0.05 for every statistical test.

## Results

### Field Efficacy of Biologically-Based Treatments

In the “CHS” vineyard cv. Semillon, a highly susceptible white cultivar, the two experimental INRA strains *E. cowanii* S22 and *B. ginsengihumi* S38 were tested in the 2015 season (Table 2). The untreated control presented 51.9% incidence and 13.4% severity of BBR. Despite application of the bacterial strains five times in the season, none of the treatments significantly reduced the incidence or severity of BBR (*p* = 0.106 for incidence, and *p* = 0.300 for severity). Severity reductions by some treatments were noticeable, ranging from 20 to 54% for the full season treatments. Moreover, the strategy based on a single application at “D” stage reduced BBR severity by 59%. Considering these remarkable differences, the lack of statistical significance clearly indicated a very high variability among the replicate plots.

In 2016, in the “CHS” vineyard (Table 2), only *B. ginsengihumi* S38 was tested, and this was done by comparing treatments that included: (i) the S38 with 33% of culture supernatant in the mixture as previously described, with or without the Sticman® adjuvant (“S38” and “S38(NoADJ)” treatments, respectively), and (ii) treatments without culture supernatant (NS: No Supernatant), with or without the adjuvant (“S38 (NS)” and “S38 (NS-NoADJ),” respectively). The untreated control presented 75.3% incidence and 20.3% severity of BBR. No significant reductions of Incidence or severity were observed (*p* = 0.315 and *p* = 0.3278, respectively). However, a clear trend of efficacy was evident for all treatments based on the S38 strain, leading to severity reductions ranging from 9 up to 49%. This interesting trend was explored by a further statistical analysis of the dataset. Since no significant effect of treatments on the BBR severity was observed with the presence of the culture supernatant, data from the “S38 (NoADJ)” and “S38 (NS-NoADJ)” treatments were pooled. Similarly, data from the “S38” and “S38 (NS)” treatments were also pooled. This pretreatment of incidence and severity means allowed us to analyze the data using a one-way ANOVA based on the presence (vs. absence) of the bacterial strain, *B. ginsengihumi* S38. The resulting significant ANOVA model showed that the presence of the BCA S38 strain decreased significantly the BBR severity (*p* = 0.049). Accordingly, the Tukey test separated means from treatments without S38 (21.0% severity) from those including the S38 BCA strain (13.7% severity). This difference represents an overall reduction of 35% in the severity of BBR in our Semillon vineyard in 2016.

In the “GF” experimental vineyard in 2016, relatively low BBR incidence and severity were observed in the untreated control, 53.4 and 4.3%, respectively (Table 3). The “Full season” strategy did not show any significant effect, nor did the “Early season” or the DRI strategies (*p* = 0.184 and *p* = 0.313 for Incidence and severity tests, respectively). Only four treatments showed, only as a trend, a reduction in severity ranging from 30% (“S38”) to 42% (“ES-S38”). Here, again, considering differences between the mean BBR intensity levels, the lack of statistical significance clearly indicated a very high variability in data.

In 2017, in the same field site “GF,” the untreated control presented 56.7% incidence and 9.9% severity of BBR (Table 3). The severity of the BBR was significantly affected by treatments (*p* = 0.042), whereas no significant differences were observed in the BBR incidence. The two “Full season” treatments with *B. ginsengihumi* S38, namely, “S38” and “S38 (NS),” achieved significant reductions in severity of 51% and 60%, respectively, compared to the control.

Concerning the trials in the IFV-CA33 experimental platform, in the 2015 season, the incidence and severity in the untreated control reached 73.0 and 16.7%, respectively, in the “Montagne” vineyard, and 72.0 and 18.4%, respectively, in the “Avensan” vineyard. In the “Montagne” plot, significant differences were detected in the BBR incidence at harvest on 22 Sept 2015 (*p* = 0.047; Table 4). The “*B. amyloliquefaciens*” and the “*B. subtilis* SM” treatments presented significantly lower incidence than the control. These two treatments reduced severity by 37 and 54%, respectively. All other BCA treatments in the first “Montagne” plot also reduced BBR severity, although not significantly (severity reductions ranging from 29 to 54%). The “Avensan” data did not show any significant results (*p*-values amounting to 0.662 and 0.73 for incidence and severity, respectively). However, except for “*Trichoderma* spp.,” most of the BCA treatments tended to reduce severity.

**Table 4:** *Botrytis* bunch rot control strategies evaluated at two experimental platforms (“Montagne,” “Avensan” and “StYzan” vineyards) including phenological stages, application dates and efficacy results.

**Strategy**	**Treatment^a^**	**A 10% flowering**	**A+ 100% flowering**	**B Pre bunch closure**	**C 10% Veraison**	**C+ Stage C + 21 days**	**D 21 days before harvest**		**% Incidence BBR^c^**	**% Severity BBR**	**% Severity reduction compared to control**	**% Incidence BBR**	**% Severity BBR**	**% Severity reduction compared to control**
**HARVEST**														
2015		M: 04/06^b^ Av: 08/06	M: 18/06 Av: 18/06	M: 03/07 Av: 07/07	M: 29/07 Av: 04/08	M: 19/08 Av:17/08	M: 03/09 Av: 28/08		**Montagne** (22/09/15)			**Avensan** (22/9/15)		
								*p*-value^d^	0.047^*^	0.158		0.662	0.730	
Untreated	Control	—	—	—	—	—	—		73.0a	16.7		72.0	18.4	
Full season	*B. amyloliquefaciens*	+	+	+	+	+	+		53.0bc	10.4	37	62.0	11.14	39
	*Trichoderma* spp.	+	+	+	+	+	+		64.0abc	11.8	29	79.9	19.2	−4
	*A. pullulans*	+	+	+	+	+	+		67.5ab	9.1	46	68.8	9.6	48
	*B. subtilis* SM	+	+	+	+	+	+		48.0c	7.7	54	69.0	15.3	17
Late season DRI based	*A. pullulans*- DRI	—	—	—	*A. pullulans*- DRI output Montagne = 3 applications Avensan = 3 applications				59.5abc	9.5	43	67.0	15.4	16
2016		M: 03/06 SY: 08/06	M: 16/06 SY:17/06	M: 12/07 SY: 13/07	M: 10/08 SY: 11/08	M: 29/08 SY: 02/09	M: 13/09 SY: 13/09		**Montagne** (04/10/16)			**St Yzan** (03/10)		
								*p*-value	0.187	0.020^*^		0.799	0.791	
Untreated	Control	—	—	—	—	—	—		82.0	12.8a		62.8	8.8	
Full season	*B. subtilis* SM	+	+	+	+	+	+		76.0	8.7bc	21	65.2	12.5	−43
	*Bacillus* sp. IP	+	+	+	+	+	+		79.2	9.2abc	18	ND	ND	ND
	*B. amyloliquefaciens*	+	+	+	+	+	+		ND	ND	ND	61.2	11.4	−30
	*A. pullulans*	+	+	+	+	+	+		68.0	7.0bc	37	63.2	10.5	−20
	*C. sake*	+	+	+	+	+	+		67.6	6.1c	45	69.2	10.7	−22
	*B. subtilis* SPS	+	+	+	+	+	+		77.6	8.4bc	25	64.8	10.2	−17
	BZ Strategy	+	+	+	+	+	+		66.8	5.9c	47	74.4	13.9	−59
Late season DRI based	*A. pullulans*- DRI				*A. pullulans*- DRI output Montagne = 3 applications St Yzan = 3 applications				81.6	10.6ab	5	70.0	10.9	−24

a *Treatments consisted of spray applications (+) of biological control agents at five or six key stages of grapevine phenology (“Full Season” strategy), unless other strategy is defined for a particular treatment. Control, Untreated; DRI, Applications following a modeled Disease Risk Index based used to trigger sprays after veraison; B. amyloliquefaciens, Amylo-X® product at 2,5 kg/ha; Trichoderma spp., Trichoderma sp. IP strain at 2.5 kg ha^−1^ A. pullulans, Botector® product at 0.4 kg ha^−1^; B. subtilis SM, Serenade Max® at 2 kg ha^−1^; C. sake, Candida sake CPA-1 at 3-4 x 10^7^ CFU ml^−1^; B. subtilis SPS, Fungisei® 3 l ha^−1^; BZ Strategy, 2 applications of Botryzen® product at 4 kg ha^−1^ (A and A+ stages) + 1 applications of MidiZen® product at 3 % (v/v) (B stage) + 2 or 3 applications of ArmourZen® product at 1 % (v/v) (C, C+ and D stages) depending of the field site*.

b *Spray application dates. “M”, “Montagne vineyard; Av, “Avensan” vineyard; “SY”, “StYzan” vineyard. Applications were carried out with a motorized back sprayer at 400 l ha^−1^ water rate*.

c *Incidence and severity of Botrytis bunch rot (BBR) were visually assessed at commercial harvest dates. Values are means of five replicate plots*.

d *p-values correspond to the “Treatment” effect in multifactorial ANOVA*.

* *Significant differences detected; Mean values connected by same letters are not significantly different (p < 0.05)*.

In the 2016 season (Table 4), the untreated control presented 82.0% incidence and 12.8% severity of BBR at the “Montagne” site (4 Oct 2016). At the “StYzan” field site, the mean incidence and severity in the untreated control amounted to 62.8 and 8.8%, respectively. Significant differences were observed in the severity of BBR at the “Montagne” location (*p* = 0.020). Five treatments out of seven significantly reduced the BBR severity by 21% (“*B. subtilis* SM”) to 47% (“BZ Strategy”). These five treatments included the commercial strains of *B. subtilis, A. Pullulans*, and *U. oudemansii*, as well as the developmental product based on *C. sake* CPA-1. In the “StYzan” field site, the results were not significant (*p* = 0.79) due to high variability, and most of the treatments showed similar, or even higher, incidence and severity values compared to the control.

In 2017, the ANOVA of the results from both field sites together showed no significant interaction between the two main factors, “Field site” and “Treatment.” Therefore, the data were pooled, and the treatment effects are summarized in Table 5. In the control, average values of pooled data were 73.6% incidence and 14.7% severity. These data emerge from incidence and severity in the control of each single field site, amounting to 79.2 and 11.5% in the “Langoiran” vineyard, and to 68.0 and 17.9% in “St Yzan” vineyard, respectively. Significant reductions in BBR incidence (*p* = 0.011) by the applied treatments were detected for “*B.subtilis* SM” and “*B. amyloliquefaciens*” (Table 5). However, all the BCA treatments significantly reduced (*p* = 0.014) the BBR severity by 31% (“S38–600 jet”) to 58% (“*B. amyloliquefaciens*”). Therefore, the reductions where therefore significant for all the treatments including the INRA bacterial strain *B. ginsengihumi* S38, as well as for the treatments based on late season applications following the modeled DRI.

**Table 5:** *Botrytis* bunch rot control strategies evaluated at two experimental platforms (“Montagne” and “StYzan” vineyards) including phenological stages, application dates and efficacy results.

**Strategy**	**Treatment^a^**	**A 10% flowering**	**A+ 100% flowering**	**B Pre bunch closure**	**C 10% veraison**	**D 21 days before harvest**		**% Incidence BBR^c^**	**% Severity BBR**	**% Severity reduction compared to control**
2017		L: 02/06^b^ SY: 02/06	L: 12/06 SY: 12/06	L: 05/07 SY:07/07	L: 26/07 SY: 27/07	L: 24/08 SY:24/08		**Pooled data: Langoiran** (22/09) **St Yzan** (28/09)		
							*p*-value^d^	0.011^*^	0.014^*^	
Untreated	Control	–	–	–	–	–		73.6a	14.7a	
Full season	*B.subtilis* SM	+	+	+	+	+		58.0bc	7.9b	46
	*B. amyloliquefaciens*	+	+	+	+	+		47.6c	6.2b	58
	*C. sake*	+	+	+	+	+		62.6ab	8.6b	41
	S38 – 400 pneumatic	+	+	+	+	+		62.0ab	7.4b	50
	S38 – 600 jet	+	+	+	+	+		67.9ab	10.1b	31
Late season DRI based	S38 – 600 jet DRI	+	+	+	+	+		63.2ab	9.7b	34
	*B. amyloliquefaciens*- DRI	–	–	–	*B. amyloliquefaciens*- DRI output Langoiran = 3 applications St Yzan = 4 applications			62.6ab	9.1b	38

a *Treatments consisted of spray applications (+) of biological control agents at five key stages of grapevine phenology (“Full Season” strategy), unless other strategy is defined for a particular treatment. Control, Untreated; DRI, Applications following a modeled Disease Risk Index based used to trigger sprays after veraison; B. subtilis SM, Serenade Max® at 2 kg ha^−1^; B. amyloliquefaciens, Amylo-X® product at 2,5 kg/ha; C. sake, Candida sake CPA-1 at 3-4 x 10^7^ CFU ml^−1^; S38, Bacillus ginsengihumi S38 strain at 5 x 10^7^ CFU ml^−1^ + Fungicover® natural product at 1 % (v/v); 400 pneumatic, applications using a pneumatic sprayer at 400 l ha^−1^ water rate; 600 jet, applications using a jet sprayer at 600 l ha^−1^ water rate*.

b *Spray application dates. Applications were carried out with a motorized back sprayer at 400 l ha^−1^ water rate, except for the “600 jet” treatments*.

c *Incidence and severity of Botrytis bunch rot (BBR) were visually assessed at commercial harvest dates. Values are means of five replicate plots per field site. Data from “Langoiran” and “StYzan” field sites were pooled prior to ANOVA, since non-significant interaction was detected between “field site” and “treatment” factors in first multifactorial analysis*.

d *p-values correspond to the “Treatment” effect in multifactorial ANOVA*.

* *Significant differences detected; Mean values connected by same letters are not significantly different (p < 0.05)*.

### Population Dynamics of Experimental INRA Bacterial Strains

**Figure 1:**
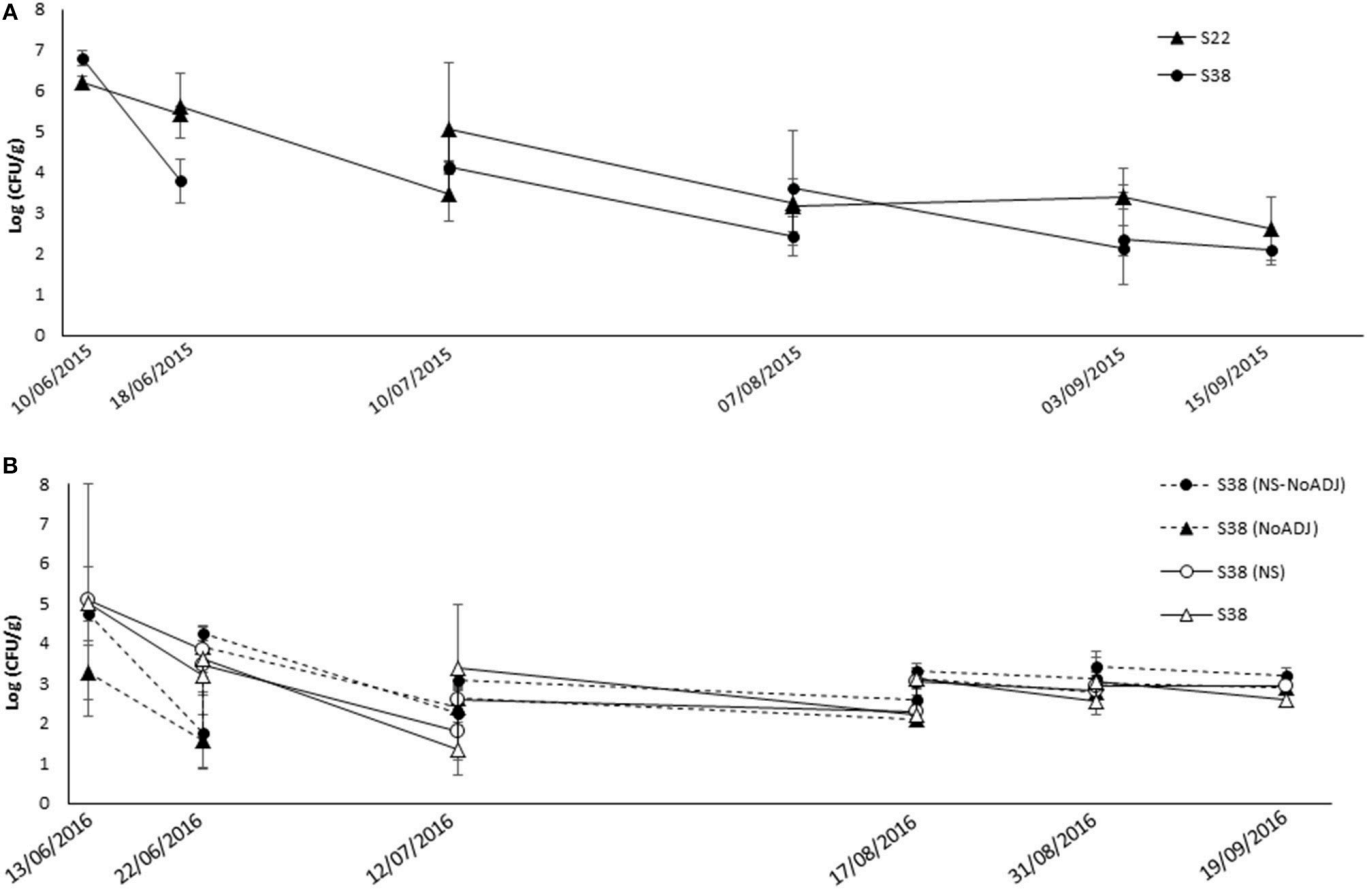
Population dynamics of experimental bacterial strains following spray applications against *Botrytis* bunch rot in the “CHS” vineyard. During the 2015 **(A)** and 2016 **(B)** growing seasons, grapevines cv. Semillon were sprayed with bacterial suspensions of **S38** (black triangles): *Bacillus ginsengihumi* S38 strain at 5 x 10^7^ CFU ml^−1^ + Sticman® adjuvant at 0.14% (v/v); or **S22** (black circles): *Enterobacter cowanii* S22 strain at 5 x 10^7^ CFU ml^−1^ + Sticman® adjuvant at 0.14% (v/v). **(NS)**: Non Supernatant in the bacterial formulation; **NoADJ**: No Sticman® adjuvant in the treatment mixture. Applications were carried out at five key phenological stages in the season: Flowering (10% cap fall; BBCH = 61); Flowering (100% cap fall; BBCH = 69); Pre-bunch closure (BBCH = 77); 10% Veraison (BBCH = 81); and 21 days before harvest (BBCH = 85). Samples of flowers and berries were collected after and just before each spray application, and at harvest date. Error bars represent Standard Deviation of the mean.

Figure 1 summarizes the evolution of bacterial populations in the CHS vineyard experiments. In 2015 (Figure 1A), *E. cowanii* S22 and *B. ginsengihumi* S38 populations maintained similar population levels before and after the spray applications. Population recovery showed very high levels (between 6 and 7 log units for both strains) after the first flowering spray (9 June 2015). Lower populations were detected at the end of flowering (mid-June). After pre-bunch closure spraying (mid-July), BCA populations in developing herbaceous berries were ~4 to 5 log units, whereas populations in ripening berries ranged from log 2 to log 4. In general, the *E. cowanii* S22 strain tended to present higher populations at most of the sampling times. However, the *B. ginsengihumi* S38 strain populations were significantly lower (*p* < 0.05) than those of the *E. cowanii* S22 only at the second sampling time (mid-June), i.e., just before the “100% flowering” application.

In 2016 (Figure 1B), populations of all *B. ginsengihumi* S38 treatments after the first spray, at 10% flowering, were ~5 log units, except for the “S38” treatment, which presented populations of 3.3 log. This difference was not significant due to the high variability of the data. Before the second spray, the two treatments that did not include the Sticman adjuvant, i.e., “S38” and “S38 (NS),” showed significantly lower population levels than the two other treatments. The Tukey test (*p* = 0.002) separated the “S38+ADJ” from the “S38” and “S38 (NS)” treatments. After this sampling time, several interesting significant differences were detected, before the “C” and “C+” sprays and at harvest (*p* = 0.002, *p* = 0.027 and *p* = 0.002, respectively) between populations in the “S38 (NS)” and in the “S38+ADJ” plots. Overall, *B. ginsengihumi* S38 populations were stable at ~3 log units in developing and ripening berries, from mid-July to the end of the season.

**Figure 2:**
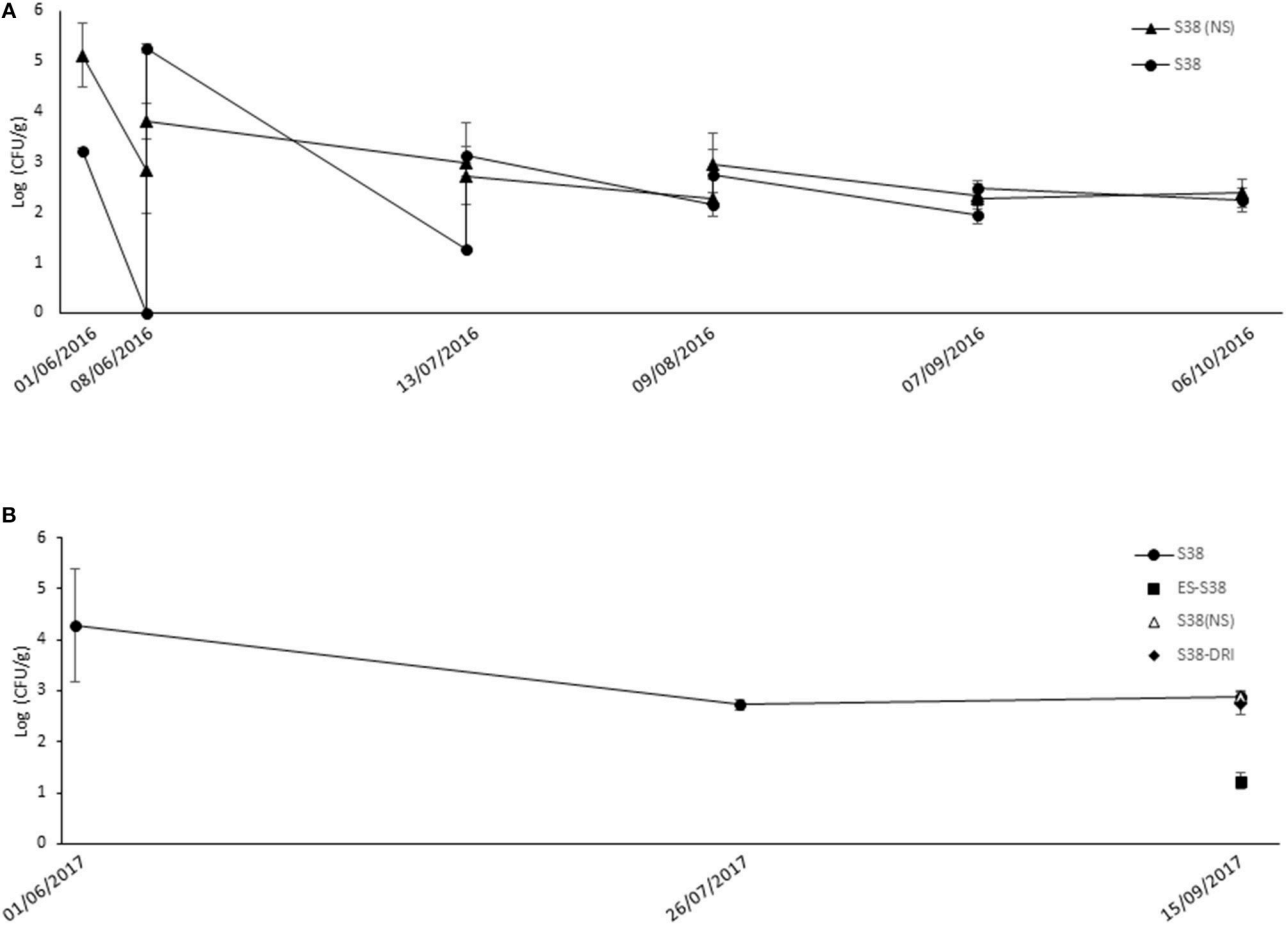
Population dynamics of experimental bacterial strains following spray applications against *Botrytis* bunch rot in “GF” vineyard. During the 2016 **(A)** and the 2017 **(B)** growing season, grapevines cv. Merlot were sprayed with bacterial suspensions of **S38**: *Bacillus ginsengihumi* S38 strain at 5 x 10^7^ CFU ml^−1^ + Fungicover® adjuvant at 1 % (v/v); **(NS)**: Non Supernatant in the bacterial formulation. Applications were carried out at five key phenological stages in the season: Flowering (10 % cap fall); Flowering (100 % cap fall); Pre-bunch closure; 10% Veraison; and 21 days before harvest. **ES**, “Early Season” strategy, only three applications from flowering to pre-bunch closure; **DRI**, Applications following a modeled Disease Risk Index used to trigger sprays after veraison (3 applications in 2017). In 2016, samples of flowers and berries for population recovery were collected after and just before each spray application, and at harvest date. In 2016, populations of S38 were assessed after the 100% cap fall and véraison sprays, and at harvest. Populations in the “ES,” “(NS),” and “DRI” treatments were only assessed at harvest. Error bars represent Standard Deviation of the mean.

In the GF vineyard experiments, only the populations of *B. ginsengihumi* S38 were monitored. In the 2016 season (Figure 2A), the populations after the first flowering application (end of May) were significantly higher when the spray formulation did not include the supernatant (5.1 log in “S38 (NS)” compared to 3.2 log in the “S38” treatment; *p* = 0.003). The inverse significant difference in populations between the two treatments was noticed before and after the second flowering spray (5.2 log in “S38” compared to 3.8 log in the “S38 (NS)” treatment; *p* = 0.005). However, populations in the “S38” treatment markedly decreased (under the “S38 (NS)” level) before pre-bunch closure. Populations in both treatments were similar after this point in the growing season, up to the last harvest sample at the beginning of October 2016.

Figure 2B shows the populations of *B. ginsengihumi* S38 in the GF vineyard during the 2017 season. The populations were assessed only at three sampling points. After the second flowering application (mid-June), population counts exceeded 4 log units. Then, the population level decreased below 3 log units after the veraison application (end of July) and remained stable until the harvest assessment. At harvest, we evaluated the plots of the three treatments “S38 (NS),” “DRI-S38” and “ES-S38,” which consisted of three applications before veraison. At this sampling time, populations of the “ES-S38” treatment (1.23 log units) were significantly lower than those of the other treatments.

## Discussion

Botrytis bunch rot represents a major threat for winegrowers, particularly near harvest time, when the application of synthetic fungicides may result in high residue levels. Reducing BBR losses in vineyards still represents a challenge for biologically based products. Microbial antagonists are one of the alternative control strategies with higher potential. However, efficacy may vary among years, climatic regions and grape varieties (Nicot et al., 2011). Our study provides a comparison of most of the available commercial biocontrol products and BCAs in the developmental stage. This study encompasses three seasons in characteristic terroirs of the Bordeaux region, in terms of grapevine cultivars and soils, to single out the best candidate antagonists for BBR in the Bordeaux to be included in complex IPM strategies in the future.

The disease pressure was variable depending on the vineyard site and the season. Severity levels in the untreated controls ranged from 4 to 20%, and exceeded 10% in most of the experiments, allowing us to observe differences among treatments with a higher consistency. In the 2015 and 2017 seasons, the BBR pressure was medium, with frequent rain episodes late in the season (data not shown) and an associated progressive disease development. These conditions favored more homogeneous incidence and severity levels at harvest among the field sites. However, in 2016, very low rainfall occurred after veraison and winegrowers postponed the harvest date to take advantage of favorable conditions for increasing the fruit ripening as much as possible. The BBR developed slowly and more irregularly with a late increase of severity in the last weeks due to very high maturity levels, which is an important factor of fruit susceptibility (Mundy and Beresford, 2007; Fuente et al., 2018). These epidemiological conditions generated the highest variability in BBR levels among the sites in the same season, from 4.3% severity in “GF” to 20.3% in “CHS.” This difference also may be explained by the severe downy mildew attack in “GF,” which reduced bunch compactness, as confirmed by the negative correlation observed between downy mildew and BBR incidence percentages (*p* < 0.0001, R^2^ = 0.48; data not shown). Furthermore, the BBR severity was also very high in “CHS” because of (i) the high susceptibility of cv. Semillon (Fuente et al., 2018) and (ii) the vineyard being near a stream, which provided higher RH levels conducive to *B. cinerea* development in a context of no rainfall. Despite the high variability in the 2016 results, overall comparisons may be appropriate among seasons and field sites.

During the three seasons, only the *B. ginsengihumi* S38 strain, among the experimental INRA candidate strains tested in present study, was able to significantly reduce BBR incidence or severity. The observed rates of reduction in the severity were achieved under considerable disease pressure conditions with significant results in four out of six site^*^season trials. Five applications of the *B. ginsengihumi* S38 strain in the “Full season” strategy were able to reduce severity by 35 to 60% compared to the untreated control. Moreover, the average significant efficacy of the “Full season” S38 treatments was 45%, which represents a very high level for a non-formulated BCA used only as a fresh cell suspension. Other studies with *Bacillus* sp. BCAs at a similar developmental stage have also shown moderate efficacy in the field (Aziz et al., 2016; Haidar et al., 2016b). However, these reduction levels in our trials are more comparable to those levels achieved with commercial products (Pertot et al., 2017b; Rotolo et al., 2018), as it will be discussed below.

In 2015 at the “CHS” field site, *B. ginsengihumi* S38 did not show any significant reduction. In this trial, the results were remarkably variable, as shown by the high (although non-significant) reductions in severity achieved by one single application of S38 (“Single D-S38”) or by the Sticman adjuvant (“ADJ”). Despite this result, five applications of S38 also reduced BBR by 71% in the “GF” field trial during 2015, as recently published by Calvo-Garrido et al. (2018). This reduction in BBR was supported by overall efficacy of the S38 observed in the present study during the subsequent years.

The variations in the preparation of the treatment mixture included the use of two different additives and the presence of cell culture supernatant. Culture supernatant accumulates the byproducts of bacterial multiplication, with some showing antimicrobial activity (Haidar et al., 2016b). The *B. ginsengihumi* S38 strain has an antibiosis mode of action and significantly inhibits *B. cinerea* germination in aqueous medium, as has been clearly demonstrated (Calvo-Garrido et al., 2018). Numerous examples of *in vitro* cell-free supernatant activity on *B. cinerea* are cited in the literature (Arroyave-Toro et al., 2017; Calvo et al., 2017; Wallace et al., 2018), however, in our field studies, the addition of the supernatant did not significantly improve the bacterial performance in the field. Furthermore, in the 2016 trials at the “GF” and “CHS” vineyards, we noticed lower S38 cell concentration and persistence on grape flowers and berries when treated with supernatant. Thus, although these differences were not consistent in time, the supernatant could potentially be excluded from a future commercial formulation of this BCA. Since the S38 mode of action include the production of antifungal compounds, the S38 efficacy may be explained by the high production of these metabolites following the field application of the BCA cells, rather than by the metabolites during bacterial production. Other reports of low efficacy of BCA culture supernatant are also reported in the literature (Long et al., 2005; Yánez-Mendizábal et al., 2011; Csutak et al., 2013; Calvo et al., 2017) suggesting cell-related mechanisms. Nonetheless, specific studies must be conducted on the S38 strain to test the antifungal activity of the supernatant and to determine the concentration and the specific composition of the antifungal molecules.

The use of adjuvants to improve cell adherence and persistence of BCAs on grapevine tissues has been justified by numerous studies (Droby et al., 2003; Qin et al., 2006; Larena et al., 2010; Calvo-Garrido et al., 2014c). This was also highlighted in our study, because populations during flowering were larger when Sticman® was included in the mixture in 2016 at the “CHS” vineyard. The use of the additive Fungicover® was preferred because the use of Sticman® in 2015 produced an odd effect favoring sour rot development in a field trial (Calvo-Garrido et al., 2018). The Fungicover® additive has been reported to show a direct anti-*Botrytis* activity (Calvo-Garrido et al., 2014a), but this effect was very limited at the 1% concentration used, as demonstrated by the non-significant effect of the “ADJ” treatments in the “GF” experiments. This compound may have accounted for maintaining high population levels when evenly distributed on flowers and berries, at least at the same levels as Sticman®. Future applications of INRA bacterial BCAs may also include this product as a generic additive, although further studies with other additives still need to be conducted.

None of the other four INRA candidate bacterial BCA tested (*Paenibacillus sp*. S18; *E. cowanii* S22, *Enterobacter* sp. S23 and *P. agglomerans* S6) showed any significant antagonistic effect on BBR. These strains were applied for the first time in this study, as they had been pre-selected with *in vivo* tests based on their improved survival ability and high efficacy (Calvo-Garrido et al., 2018). *P. agglomerans* S6 was only tested in one season, but it is unlikely to be effective in field conditions since S38 had a very clear effect in the same experiment. *Paenibacillus* sp. S18 and *Enterobacter* sp. S23 were also tested only in one experiment where no significant result was obtained (“GF” in 2016). Similarly, the combination of the S18 and S23 strains with *B. ginsengihumi* S38 (“Cocktail” treatment) did not achieve any reduction under the 2016 irregular experimental conditions. The *E. cowanii* S22 strain showed no effect in the previously published 2015 trial in “GF”(Calvo-Garrido et al., 2018) and a low biocontrol capacity in our Semillon vineyard trial during the same season. Hence, this result confirmed low interest for the use of this strain in future trials. The lack of efficacy of *E. cowanii* S22 is not in accordance with its high population levels observed during the entire 2015 season. Similarly, high populations of S38 were counted at harvest in the “S38” and the “DRI-S38” treatments in 2017; however, the latter treatment had a much lower efficacy. These facts highlight the complexity of the factors influencing the field efficacy of biological control, most notably against BBR. High populations are required for effective control, but other factors may also be important. For example, S22 showed very high *in vivo* efficacy and high remaining populations in the field; however, other conditions such as water activity, optimal temperature for antibiosis or UV radiation may constrain the antagonistic activity of BCAs (Magan, 2001; Lahlali et al., 2011; Pertot et al., 2012; Haidar et al., 2016b). Identifying these factors constitutes a key factor for improving the performance of BCAs, in particular the INRA strains. When the efficacy of commercial BCA formulations, or those close to registration, are considered, most of the products achieved a certain reduction of BBR. However, the results were not always significant. Four of the antagonists were tested only in 1 year of experiments: *Trichoderma* spp.*, Bacillus* sp. IP, *B. subtilis* IAB/BS03, and *U. oudemansii*. Only two treatments with these BCAs were effective and can represent a potential control strategy for the Bordeaux conditions, i.e., *U. oudemansii* and *B. subtilis* IAB/BS03. Most of the consistent results corresponded, however, to the products included in two or more seasons. Although registered showing efficacy under certain conditions (Pertot et al., 2017b), the *A. pullulans* formulation significantly reduced the BBR in only one out of four site^*^year trials. Therefore, this BCA formulation does not seem suitable for the conditions tested, which is supported by previous results in the Bordeaux area (Calvo-Garrido et al., 2017a). However, the *B. subtilis* QST713, *B. amyloliquefaciens* and *C. sake* strains significantly reduced severity in 4/6, 3/5, and 3/4, respectively, of the site^*^year trials in which they were included. For some of the formulations, the rates in reduction achieved were relatively high compared to other results reported in the literature. For example, moderate severity reductions in the field, from 5 to 35% were detected by Rotolo et al. (2018), whereas other studies have reported no significant differences (Mehofer et al., 2009; Bay et al., 2010). Nonetheless, Pertot et al. (2017b) observed 80–95 % reductions in severity in several field trials in north Italy, and other reports of positive results have been published worldwide (Schilder et al., 2002; Elmer and Reglinski, 2006; Thomidis et al., 2016). In Northeastern Spain, two studies in recent years have evaluated field treatments using different formulations of *C. sake* CPA-1, and these studies achieved similar reductions to those observed in our experiment (Calvo-Garrido et al., 2017b; Carbó et al., 2018). Data concerning *B. amyloliquefaciens* in vineyards are scarce in the literature because the product has only recently entered the market. Manufacturer references claim up to 90% efficacy, although a recent study in table grapes showed no efficacy after up to 11 sprayings (Rotolo et al., 2018). Overall, these three BCAs represent good candidates for optimizing and increasing BCA use in vineyards and for reducing the number of conventional anti-*Botrytis* sprays, as well as for including them in IPM strategies. The combination of BCAs with other BCAs or with low risk NPs can consistently reduce BBR in the field (Calvo-Garrido et al., 2013; Pertot et al., 2017b; Rotolo et al., 2018). Designing new complex strategies that combine several of these BCAs, with a few applications focused at key moments is the next step in research to achieve reliable BBR control programs under the conditions found in the oceanic Bordeaux region, as well as in other viticultural areas conducive to BBR. In this sense, this work was based on several principles of IPM, by developing new BCAs, combining tactics and product modes of action, evaluating multi-season effects and improving the decision-making process (Barzman et al., 2015).

According to present results, different modes of action of the BCAs accounted for effective control of BBR. Most of the *Bacillus* sp. strains (*B. amyloliquefaciens, B. subtilis* QST713, *Bacillus subtilis* IAB/BS03, and *B. ginsengihumi* S38), achieved consistent efficacy in our trials with antibiosis as their major mode of action, indicating that this mode of action has a high potential for *B. cinerea* control in vineyards. Nevertheless, two strains with reported antibiosis as mode of action, although in a very early developmental stage as BCAs, did not shown any effect (*E. cowanii* S22 and *B. subtilis* IP). Regarding the strains with nutrient competition as major mode of action, *C. sake* was consistently effective but *A. pullullans* was not. These facts indicate that antibiosis may be a more suitable mode of action in the conditions tested and for the BCA list that we included. However, the variability shown by different strains with similar modes of action suggests that efficacy depends also on other strain features, and hence the objective may be to identify the reliable BCAs in a region and try to combine their different modes of action. In our case, for instance, an interesting spray program will include *C. sake* at flowering and at veraison, to provide a preventive protection of infections, due to nutrient deployment and good survival, combined with *Bacillus* sp. strain sprays during fruit ripening, when a curative action (antibiosis) is desired to stop symptom progression.

Throughout the three seasons, a reduction in BBR was achieved by applying the antagonists five or six times during the season, which can be considered excessive in terms of technical and economic effort for growers. In this study, we also investigated the possible reduction in the number of applications, hence diminishing the treatment strategy cost. Preventing *B. cinerea* infection in flowers and reducing secondary inoculum sources is considered an effective strategy for reducing BBR at harvest (Nair et al., 1995; Calvo-Garrido et al., 2014b; Fedele et al., 2017). In this study, however, three applications of *B. ginsengihumi* S38 before veraison were not effective. Though, a 20% reduction in the 2017 season, under medium disease pressure, highlighted interest in applying the S38 strain at flowering under particular year conditions that remain to be further characterized.

The application of BCAs, based on BBR risk after veraison was only effective in the 2017 trial. Under those experimental conditions, applications of *B. amyloliquefaciens* and *B. ginsengihumi* S38, following the DRI output, achieved similar reductions to five applications of the “Full season” strategy. Moreover, the number of DRI applications was low in the very low disease pressure situation (“GF” in 2016), confirming a good sensitivity of the DRI to conditions with low epidemiological risks. However, the adjustment of the model's sensitivity to higher risk levels is still unsatisfactory. The DRI model output suggested numerous risk alerts that resulted in three to four applications in most of the case studies. This adjustment of decision rules according to the fruit maturation process, corresponding to the model's adaptation to ontogenic resistance of each cultivar (Deytieux-Belleau et al., 2009; González-Domínguez et al., 2015), constitutes the main future research focus in the application of the DRI developed and used in this study. Other researchers are also working to develop such a practical decision-support system for growers against BBR (González-Domínguez et al., 2015), which represent a long process requiring an intense effort in mathematical modeling and associated field testing.

Altogether, our vineyard trials confirmed the efficacy of *B. ginsengihumi* S38, a bacterial BCA recently developed at INRA, Bordeaux. Reduction levels in BBR by this strain were similar to those achieved by commercial BCA products throughout the same three seasons in the same important grapevine growing area. The consistency of the disease control demonstrates the high potential of this strain for future application in Bordeaux and other viticultural areas worldwide, as well as for future BCA product development. Opportunities to improve biocontrol field efficacy and consistency include optimizing the production and formulation of the BCA. Moreover, further studies will be needed that investigate combinations of this BCA with other biocontrol formulations for use in IPM strategies and to optimize application timing. Our results also offer growers a comparison of those biocontrol products that are already or soon will be on the market. Among them, three antagonists, *B. subtilis* QST713, *B. amyloliquefaciens* subsp. *plantarum* strain D747, and *C. sake* CPA-1, are of interest for their application in the Bordeaux vineyards, especially as part of novel IPM strategies that may combine these BCAs with other control strategies. These practical control solutions respond to the needs of wine consumers, as well as winegrowers, who are showing more interest in the production of high-quality wines with high safety standards, including the absence of pesticide residues in the final product.

## Author Contributions

CC-G and MF: major contribution in experimental work sensu lato, data analysis and manuscript elaboration; JR and LD: major contribution in experimental work sensu lato; NA and SD: major contribution in experimental work sensu lato and data analysis.

### Conflict of Interest Statement

The authors declare that the research was conducted in the absence of any commercial or financial relationships that could be construed as a potential conflict of interest.
